# Genomic analysis of *Caldalkalibacillus thermarum* TA2.A1 reveals aerobic alkaliphilic metabolism and evolutionary hallmarks linking alkaliphilic bacteria and plant life

**DOI:** 10.1007/s00792-020-01205-w

**Published:** 2020-10-08

**Authors:** Samuel I. de Jong, Marcel A. van den Broek, Alexander Y. Merkel, Pilar de la Torre Cortes, Falk Kalamorz, Gregory M. Cook, Mark C. M. van Loosdrecht, Duncan G. G. McMillan

**Affiliations:** 1 Department of Biotechnology, Delft University of Technology, Delft, The Netherlands; 2 Winogradsky Institute of Microbiology, Research Center of Biotechnology, Russian Academy of Sciences, Moscow, Russia; 3 The New Zealand Institute for Plant and Food Research, Lincoln, New Zealand; 4 Department of Microbiology and Immunology, The University of Otago, Dunedin, New Zealand

**Keywords:** Genome, Alkaliphiles, Phylogeny, Evolution

## Abstract

The aerobic thermoalkaliphile *Caldalkalibacillus thermarum* strain TA2.A1 is a member of a separate order of alkaliphilic bacteria closely related to the *Bacillales* order. Efforts to relate the genomic information of this evolutionary ancient organism to environmental adaptation have been thwarted by the inability to construct a complete genome. The existing draft genome is highly fragmented due to repetitive regions, and gaps between and over repetitive regions were unbridgeable. To address this, Oxford Nanopore Technology’s MinION allowed us to span these repeats through long reads, with over 6000-fold coverage. This resulted in a single 3.34 Mb circular chromosome. The profile of transporters and central metabolism gives insight into why the organism prefers glutamate over sucrose as carbon source. We propose that the deamination of glutamate allows alkalization of the immediate environment, an excellent example of how an extremophile modulates environmental conditions to suit its own requirements. Curiously, plant-like hallmark electron transfer enzymes and transporters are found throughout the genome, such as a cytochrome *b*_6_*c*_1_ complex and a CO_2_-concentrating transporter. In addition, multiple self-splicing group II intron-encoded proteins closely aligning to those of a telomerase reverse transcriptase in *Arabidopsis thaliana* were revealed. Collectively, these features suggest an evolutionary relationship to plant life.

**Electronic supplementary material:**

The online version of this article (10.1007/s00792-020-01205-w) contains supplementary material, which is available to authorized users.

## Introduction

The alkaline world is a fascinating and geologically ancient environment (Horikoshi 2016). In line with this, Russell and Hall first theorized that life on earth might well have started in alkaline hot springs (Russell et al. 1988; Russell 2006), but whether this is true for all life is still a matter of intense debate (Miller and Bada 1988; Damer and Deamer 2015; Maruyama et al. 2019). Support for this theory appears to be ‘branched’, and is built on the plant-specific processes of carbon fixation (Braakman and Smith 2012), which we will not discuss at length here, and cellular energy generation. Interestingly, the latter provides some the most substantial genetic evidence that plant life finds its origin in alkaline environments (Russell et al. 1988). The original proposal centers on electron transport via iron-sulfur clusters in a primitive ‘electron transport chain’ (ETC). In commonly studied bacteria such as *Escherichia coli*, the electron transport chain consists of an electron-donating reaction, generally either an NADH dehydrogenase (complex I) or a succinate dehydrogenase (complex II). The other reaction logically is an electron-accepting reaction, catalyzed by a terminal cytochrome oxidase (complex IV), which transfer the electrons to an oxidized compound—oxygen in case of aerobic microorganisms. In some bacteria and in all animal life, the electron donating and accepting reactions have an electron splitting reaction, a cytochrome *bc*_1_ complex (cyt.*bc*_1_; complex III) in between them, as a form of regulation and also energy generation. In bacteria and archaea, classical complexes I, III and IV can translocate H^+^ or Na^+^ over the membrane, thereby generating an ion-motive force (Mitchell 1966; Speelmans et al. 1993). The resulting gradient is harvested by the F_1_F_o_ ATP synthase in most bacteria (complex V). The soluble F_1_ domain is responsible for the catalytic activity, while the F_o_ domain is responsible for importing the translocated H^+^ or Na^+^. Translocation of protons is via the *a* subunit and a proteolipid *c*-subunit ring of organism specific size (Elston et al. 1998; Stock et al. 1999).

A clue about evolution stems from the stoichiometry of the rotating, membrane embedded, *c*-subunit ring of ATP synthase. This ring consists of a multimer of eight in *Bos taurus*, to fourteen-mer in chloroplasts and onwards to fifteen-mer in cyanobacteria. Interestingly enough, having a large *c*-ring is actually not advantageous, if purely assessing the amount of ATP produced per proton pumped. Regardless, a large *c*-ring is a common trait for alkaliphiles, cyanobacteria and plants (Pogoryelov et al. 2005; Vollmar et al. 2009; Watt et al. 2010; Nesci et al. 2016). One of the most deeply rooted member of the *Bacilli* class, the thermoalkaliphile *Caldalkalibacillus thermarum* strain TA2.A1 (Peddie et al. 1999). *C. thermarum* TA2.A1 has a large thirteen-mer *c*-subunit ring (Meier et al. 2007), and its ATP synthase has been extensively researched (Cook et al. 2003; McMillan et al. 2007, 2016). However, relatively little has been described on its preceding ETC and current genetic data is incomplete. Thus, we cannot to determine whether it contains all possible components of the ETC, which is crucial for drawing evolutionary conclusions, and for further research in general.

The NCBI database actually does state that sequencing data from 2011 constitute a representative genome. However, considering the 18 × fold 454 GS FLX (and 261 × Illumina coverage), it was actually denoted as only a high quality draft, with some small regions possibly remaining undiscovered (Kalamorz et al. 2011). The draft could not be assembled due to complexity of the repetitions present, leaving the genome scattered over 251 contigs. Repetitive regions are not unusual in a genome and can indicate multiple features. In bacteria they feature as control region for expression (Patrick et al. 2003) or as regions regulating recombination (Sekulovic et al. 2018). Another, quite noteworthy example for bacteria, is that of the CRISPR-associated regions (Mojica et al. 2000; Horvath and Barrangou 2010). All of the three aforementioned features contribute to bacterial fitness, something that is expected to be of prime importance for a polyextremophile.

Oxford Nanopore Technology’s MinION platform enables us to sequence long reads (up to 200 kb), which should resolve the problem of repetitive regions in the genome of *C. thermarum* TA2.A1. A drawback of the technique is the fact that it does have an error rate of up to 12% (Goodwin et al. 2016).Therefore, only when supplementing this technique with the precise Illumina technology will we obtain a representative genome. In this research paper, we sequenced *C. themarum* TA2.A1 using the abovementioned techniques. We use the improved genomic data to outline new features and its implications for observed physiology. We also discuss additional evolutionary perspectives this genome provides for the alkaline hot pool theory.

## Materials and methods

### Bacterial strain, growth conditions

*Caldalkalibacillus thermarum* strain TA2.A1 was cultured as described previously (McMillan et al. 2009). *C. thermarum* TA2.A1 was grown aseptically in a shaking incubator (180 rpm) at 65 °C in an alkaline basal medium containing 10 g L^−1^ trypticase peptone at pH_65 °C_ 9.5 or as indicated.

Growth was initiated with a 0.1% inoculum from an overnight culture. For DNA extraction and pH-shift studies 10 g L^−1^ l-glutamate was used as the major carbon source and cells were grown aerobically in a round bottom shake flask overnight. When l-glutamate was excluded from this medium, it was replaced with sucrose to a final concentration of 10 g L^−1^. Where necessary, growth was monitored by aseptically extraction of samples and measuring the optical density at 600 nm (OD_600_) (1-cm light path length) or measuring dry weights. Dry weights were obtained by filtration through a filter (0.2 µm Millipore) and drying thereafter in a 105 °C oven for at least 24 h.

### DNA extraction, sequencing, and assembly

DNA was isolated according to the QIAGEN^®^ Genomic DNA Handbook, from 5 mL overnight culture. Sequencing was performed with both Illumina^®^ MiSeq and Oxford Nanopore Technologies MinION platform. Genome was de novo assembled with Canu (Koren et al. 2017) and thereafter annotated with Prokka, RAST (Aziz et al. 2008; Overbeek et al. 2014; Brettin et al. 2015) and BlastKOALA (Seemann 2014; Kanehisa et al. 2016). A single annotation file was made by combining the three annotations and manually curating discrepancies between algorithms.

### Phylogenetic analysis

Protein-based phylogeny. A list of 120 bacterial core genes was taken from Genome Taxonomy DataBase (GTDB) (Parks et al. 2020). These marker genes were identified in selected genomes, aligned and concatenated using GTDBtk v0.3.2 (Chaumeil et al. 2019). Alignment was automatically trimmed using trimAl 1.2rev59 using automated1 and gt 0.95 options (Capella-Gutiérrez et al. 2009). The resulting alignment consisted of 21,432 amino acid residues. Phylogenetic tree was built using IQ-TREE 1.6.12 program (Nguyen et al. 2015) with SH-aLRT test (Anisimova et al. 2011) as well as ultrafast bootstrap with 1000 replicates (Hoang et al. 2018) and ModelFinder to determine the best-fit model (Kalyaanamoorthy et al. 2017).

For *Bacillus* sp. genome comparison, whole genome sequences were downloaded from NCBI and processed by kSNP3 (Gardner and Slezak 2010; Gardner and Hall 2013; Gardner et al. 2015). A parsimony tree was inferred by kSNP3 with default settings and a kmer of 21, which was determined by Kchooser. The resulting newick file was visualized with MEGA 8 resulting in a phylogenetic tree.

## Results and discussion

### Contempary sequencing methodologies enable the assembly of a complete circular chromosome

**Fig. 1:**
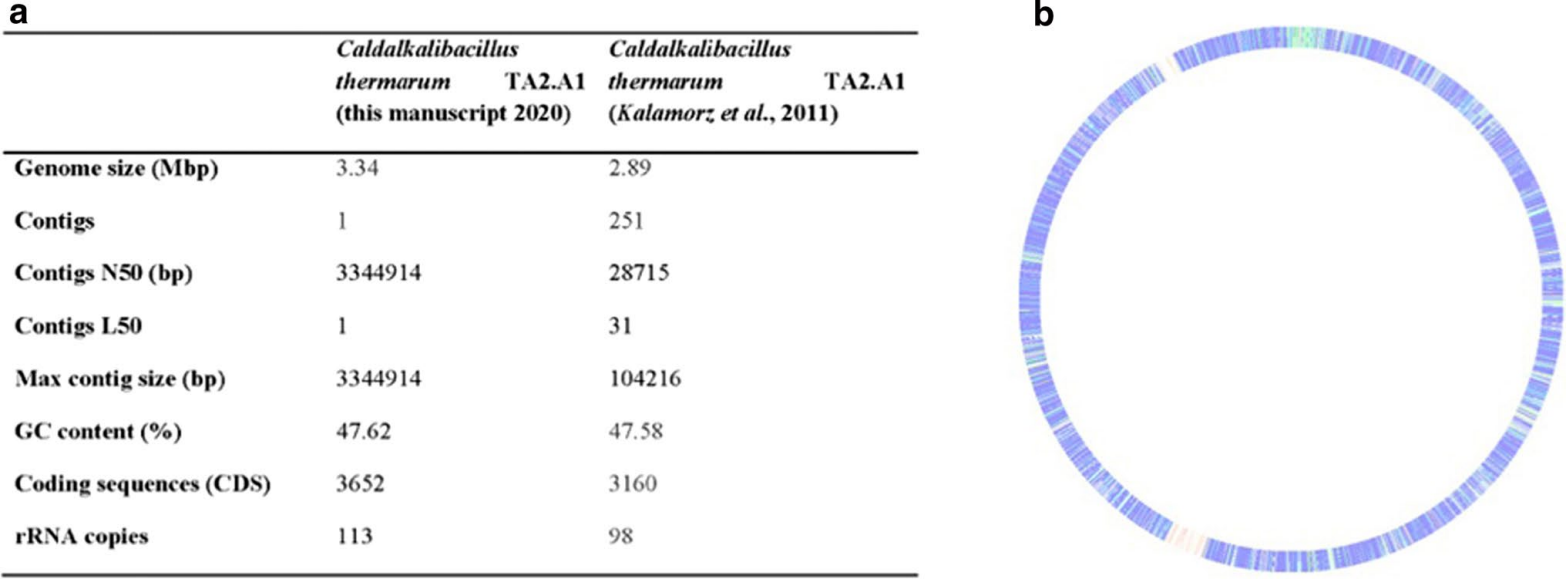
Comparison and Alignment of old and new genetic data of *Caldalkalibacillus thermarum* strainTA2.A1. **a** Statistical analysis of the assembled and annotated genome sequence of *C. thermarum* TA2.A1 from this manuscript compared to the 2011 publication ^24^. **b** The new circular genome is used a reference to which the old data is aligned. Green/blue indicates a 90–100% alignment, red/pink/absence of color indicates no sequence overlap. This alignment shows how the old, fragmented high-quality draft compares to the complete, new singular chromosome. As is visualized, some regions were represented well in the old draft, while some were not represented at all, which leads to the observed discrepancy in genome length between the two studies. The alignment was visualized using the online global alignment function of RAST (Aziz et al. 2008; Overbeek et al. 2014; Brettin et al. 2015)

MinION and Illumina sequencing resulted in 6173 × and 621 × coverage, respectively, with an average read length of 10.50 kb and 301 bp, respectively. Especially the high coverage with the MinION, when compared with the old sequencing data (Fig. 1a), aided us in spanning repeat regions present in the genome.

The successful assembly resulted in a single circular chromosome (Fig. 1b) of a total sequence length of 3.34 Mb, a 15.5% increase in length over the 2011 study. 3652 coding sequences were found, up from 3160 genes reported by deposited data in the NCBI database (Kalamorz et al. 2011). These were annotated as described above. The new, annotated *C. thermarum* TA2.A1 sequence was uploaded to the NCBI database, with accession number: PRJNA638815.

### Phylogeny

**Fig. 2:**
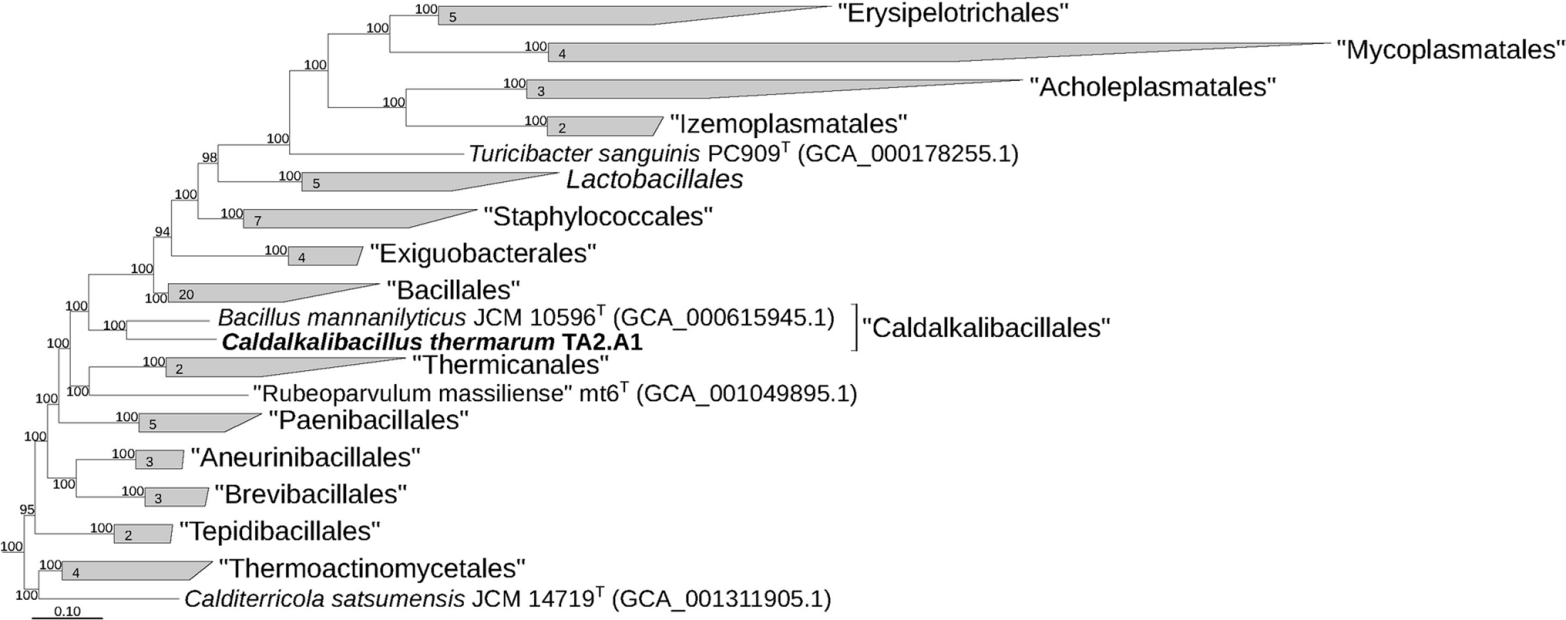
*Caldalkalibacillus thermarum* strain TA2.A1 is in a novel order “*Caldalkalibacillales*” lacement of *C. thermarum* TA2.A1 within the class *Bacilli* based on phylogenetic analysis of concatenated partial amino acid sequences of 120 bacterial conservative proteins ^40^ by maximum likelihood inference; taxonomic designations correspond with Genome Taxonomy DataBase ^40^. Bootstrap values are shown at the nodes. Bar, 0.10 changes per position

A full genome gives us the possibility of analyzing the phylogenetic relationship of *C. thermarum* TA2.A1 within the Firmicutes phylum (Fig. S1) and the *Bacilli* class (Fig. 2). For this analysis, we chose to use a core set of 120 reference genes (Parks et al. 2020) taken from the Genome Taxonomy DataBase (GTDB), with the taxonomic trees constructed as described above. The phylogenetic analysis shows that based on amino acid sequences of single copy marker genes *C. thermarum* TA2.A1 is part of the “*Caldalkalibacillales*” order, almost all by itself. The GTDB classification is in contrast with what NCBI reports, which states common ancestry with *Bacillus* species only diverges at genus level. We propose that the GTDB classification, confirmed by the findings of this study, to be the correct classification. *C. thermarum* TA2.A1 is joined in its order by *Bacillus mannanilyticus* JCM 10596^ T^, which is an mesophilic, moderately halotolerant, alkaliphilic chemoorganotroph (Nogi et al. 2005).

Based on our phylogenetic reconstruction, two representatives of the "*Caldalkalibacillales*" order with available genomes form a separate deep phylogenetic branch of the order level within the *Bacilli* class (Fig. 2). Most other known halophiles and alkaliphiles are part of “*Bacillales*” order, which curiously is the most closely related order. We also note a far more distant relationship with other (an-)aerobic thermophiles within the Firmicutes phylum (Fig. S1).

However, to further corroborate genomic information with functional data, well-characterized bacterial systems are required for comparison. In light of this limitation we decided to compare *C. thermarum* TA2.A1 with environmentally similar well-describe alkaliphilic *Bacillus* sp., the mesophilic *Bacillus* sp, and the thermophilic *Geobacillus stearothermophilus*. For this analysis, we chose to use a reference free whole genome single nuclear polymorphim (SNP) based phylogeny on *Bacillus* species. SNPs are the most common type of genetic variation and allow a rapid, but more wide reaching than classical 16S rRNA, and are frequently used as biological markers (Schürch et al. 2018). In agreement with the analysis in Fig. 2 and Figure S1, *C. thermarum* TA2.A1 is the most phylogenetically ancient member of the genus *Bacillus* examined (Fig. S2).

### Origin of replication

Initially, we attempted to find the origin of replication of *C. thermarum* TA2.A1. For related alkaliphiles such as *Bacillus halodurans* C-125 and *Bacillus pseudofirmus* OF4, ‘origin regions’ have been specified, not a *bona fide* oriC regulatory region such as that for *Escherichia coli* K-12 (Meijer et al. 1979). The oriC region is of significance, as this is the binding site of dnaA, the chromosomal replication initiation enzyme (Fuller et al. 1984), which starts DNA unwinding and subsequent loading of the replisome. The model organism in the *Bacilli* class, *Bacillus subtilis*, has a fragment of DNA denoted as an oriC sequence (accession: X02369), yet an annotated oriC region is not discretely identified. The putative oriC region roughly ends with the coding region of gyrB.

**Fig. 3:**
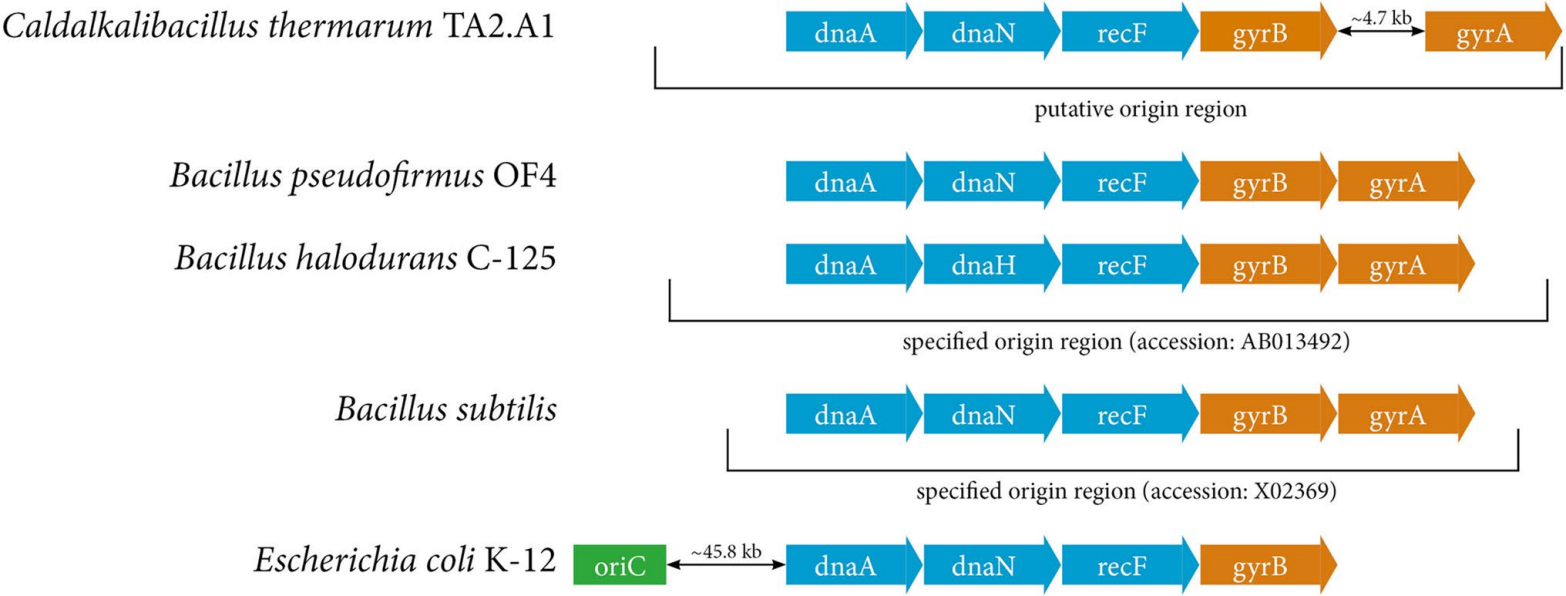
Alignment of putative origin regions of various *Bacillus* species and *E. coli*. Organisms included are *C. thermarum* TA2.A1, *B. pseudofirmus* OF4 (accession: CP001878), *B. halodurans* C-125 (accession: AB013492) and *B. subtilis* (accession X02369). The genes dnaA, dnaN/H and recF are part of the bacterial replisome, while gyrA and gyrB code for DNA gyrase, a topoisomerase, which is not related to chromosomal replication. *E. coli* K-12 (accession AP009048) is included as comparison, as this has an oriC region, which removed ~ 45.8 kb from its dnaA gene

Another putative origin of replication for a species in the *Bacilli* class, *B. halodurans* C-125 (accession: AB013492), similarly shows gyrB close to its end, and additionally shows dnaA close to its start (Fig. 3). The dnaH and dnaN genes are directly involved in the replisome (O’Donnell 2006), while DNA gyrase, a topoisomerase encoded by gyrA and gyrB, is crucial for unwinding DNA during cell replication (Zechiedrich and Cozzarelli 1995). The genome of *C. thermarum* TA2.A1 also has a region spanning from dnaA to gyrB, which could contain the origin of replication (i.e. the oriC region). We do note that in the case of *E. coli* K-12, the oriC region is over 45 kb away from the replication initiating gene dnaA (Fig. 3). The 4.7 kb space between gyrB and gyrA is seemingly unique to *C. thermarum* TA2.A1 and it contains import machinery for magnesium and molybdate, and, most crucially, the regulator cysL, which has been described to regulate the sulfite reductase operon in *B. subtilis* (Guillouard et al. 2002).

This alignment reveals a commonality exists within the *Bacilli* class regarding the origin of replication, and that a defined oriC sequence is not a commonality might indicate that this sequence is order-specific within the taxonomic class.

### Redundancy in CRISPRs, plant-like self-splicing intron-encoded proteins, and other genes

Surprisingly, a set of genes is found scattered over the genome called *ltrA* (numbered 1 to 13), which is a self-splicing group II intron-encoded protein. This caught our attention, as this is presumed to be a precursor to the eukaryotic intron mechanism. The enzyme is multifunctional, as it has reverse transcriptase activity next to maturase and endonuclease activity (Cousineau et al. 2000). This reverse transcriptase activity aligns for 53% with the TERT gene of *Arabidopsis thaliana* (query cover based on amino acid sequence). The TERT gene encodes for telomerase reverse transcriptase, a gene crucial for maintaining linear architecture in eukaryotic chromosomes. We therefore question whether this gene, or related genes, could also be an evolutionary precursor of eukaryotic telomerase machinery. The presence of *ltrA* in *Bacilli* is seemingly ubiquitous, as for most domains of life in general (Toro 2003), though we observe notably higher copy numbers in fellow alkaliphiles *B. halodurans* C-125 (5 copies) and *B. pseudofirmus* OF4 (4 copies) than in neutrophilic *B. subtilis* (2 copies) or thermophilic *Geobacillus stearothermophilus* (0 copies; accession number: PRJNA252389). Apart from the copy number, the similarity between alkaliphilic variants of *ltrA* and the TERT gene of *A. thaliana* is much higher (see above) than that of the neutrophilic *B. subtilis*, which has only 4% query cover based on amino acid sequence, meaning that in terms of evolutionary relevance, the study of an alkaliphilic *ltrA* is much more applicable.

Within the genome of *C. thermarum* TA2.A1, we find nine genes encoding for CRISPR-associated proteins; three copies of the Cas1 and Cas2 adaptation genes, two copies of Cas3 and a single copy of Cas9. The presence of two Cas3 genes suggests that *C. thermarum* TA2.A1 has two different type I systems and the single Cas9 gene indicates a single type II system. Collectively these give *C. thermarum* TA2.A1 a strong capacity for defense against phages (Pougach et al. 2010). Interestingly, while CRISPR is best known for phage resistance, Weinberger et al. have shown it is only present in ~ 45% of mesophilic bacterial genomes available on databases, whereas it is found in ~ 90% of thermophilic bacterial genomes available on databases, and are prevalent in thermophilic *Bacilli* (Weinberger et al. 2012). Mesophilic *Bacilli* have no such degeneracy as far as we are aware. The authors used models to show that as higher mutation rates in viruses increase, the rate of CRISPR spacer addition decreases. They suggest that because both mesophilic viruses and bacteria mutate more frequently that this effectively outruns CRISPR/Cas based immunity (Weinberger et al. 2012). With this in mind, the presence of three CRISPR/Cas systems in *C. thermarum* TA2.A1 suggests that phages existing in thermoalkaliphilic conditions must have relatively low mutation rates making them potentially useful molecular tools.

**Fig. 4:**
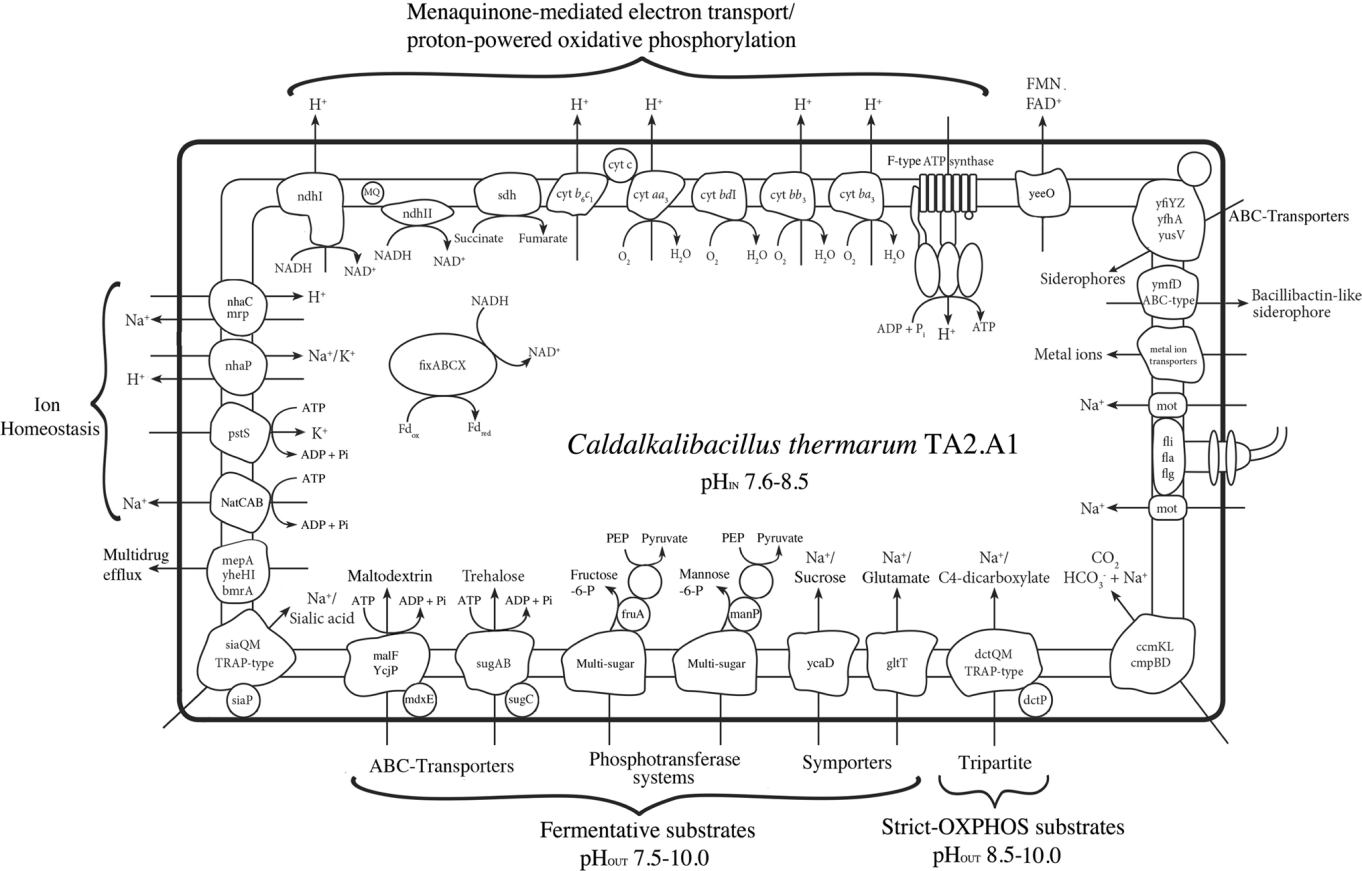
A selection of transporters and membrane proteins present in *C. thermarum* TA2.A1 based on the annotated genome. Note that two different substrate categories are present: fermentable and non-fermentable. Fermentable substrates enable *C. thermarum* TA2.A1 to adapt to a far greater variety of conditions than substrates that have to be consumed through the TCA cycle

This observed genetic redundancy is not confined to CRISPR/Cas systems. The genome of *C. thermarum* TA2.A1 contains a total of six copies of smc, the gene responsible for chromosome partitioning and at least three annotated operons dealing with spore germination, encoded by gerABC and yndE. *C. thermarum* TA2.A1 has three complete copies of pdhABCD, the operon for pyruvate dehydrogenase. The microorganism also has multiple sets of the gsiBCD genes, encoding for glutathione binding and import. Interestingly enough, only one copy of the gsiBCD genes constitute an operon, which begs the question what the other copies are for. This phenomenon is prevalent with other transporters as well; we give the C4-dicarboxylate, lactose, arabinose and trehalose importers as examples. The same incomplete duplication is something we observe for genes encoding for DNA polymerase III—some genes are present only once, while others have three copies scattered across the genome. While degeneracy of transporters are relatively common in Bacilli, such as multidrug efflux in *Bacillus subtilis* or methods of transport such as a symporter and PTS systems capable of transporting the same substrates (see Fig. 4), and metal transport (Moore and Helmann 2005), spore germination and chromosomal partitioning is usually restricted to a single operon in described Bacilli to date (Fort and Errington 1985; Lewis and Errington 1997).

### Key features of the *C. thermarum* TA2.A1 genome physiology and metabolism

Previous publications on *C. thermarum* TA2.A1 have heavily focused on particular aspects of cellular physiology in isolation, such as transporter activity (Peddie et al. 1999, 2000), cell physiology(McMillan et al. 2009), siderophore production (McMillan et al. 2010), and the F-type ATP synthase (Meier et al. 2006; McMillan et al. 2007). Here, we take the opportunity of having a holistic picture to assemble these into a thorough description by reflecting on the genome. Alkaliphiles, like most groups of highly extremophilic organisms are highly fastidious in growth, so the utmost care must be taken when cultivating them. One peculiarity of alkaliphiles is the reliance on specific types of peptone/tryptone extracts and the continued lack of ability to find a general chemically defined growth media for cultivation. It has been proposed that this is due to specific need for oligopeptides or dipeptides, yet extensive studies on alkaliphiles have not yet revealed what these are and they may indeed be organism-specific (Duckworth et al. 1996; Johnvesly and Naik 2001; Horikoshi 2004; Kevbrin 2019). *C. thermarum* TA2.A1 is no exception, while being reported to grow on a wide variety of carbon sources while remaining strictly aerobic (Fig. 4), and has a particular proclivity for growth on glutamate (McMillan et al. 2009).

### Transporters

*Caldalkalibacillus thermarum* TA2.A1 has a broad variety of substrate/sugar transporters, for (in-) organic substrates and siderophores. Most of the organic substrate uptake machinery is chemical energy dependent, ATP-utilizing ABC type (e.g. maltodextrin and trehalose) and Phophoenol pyruvate (PEP) phosphotransferase type transporter dominate (e.g. fructose and mannose) (see Fig. 4).

Interestingly, glutamate and sucrose, the substrates on which the microorganism grows best, can be imported through secondary symporter transport systems (Fig. 4), utilizing a sodium-motive force (SMF), in agreement with what has been described experimentally for both glutamate (Peddie et al. 1999) and sucrose (Peddie et al. 2000). C4-dicarboxylates, such as succinate and malate, have also been demonstrated be imported into the cell utilizing an SMF (McMillan et al. 2009), a flux which is likely controlled by a voltage-gated Na^+^ channel (Tsai et al. 2013). Interestingly we have only found a single candidate transport system capable of this function in a Tripartite (TRAP)-type transporter (Fig. 4). While symporters are mass-transport type, TRAP transporters are regarded as scavenging, indicating that while *C. thermarum* TA2.A1 is clearly capable of growth on C4-dicarboxylates (McMillan et al. 2009), it is unlikely that these are very bioavailable in an environmental setting. We also note a TRAP-type sialic acid transporter (Fig. 4). While sialic acids in bacteria were previously associated exclusively with pathogens in immune response avoidance (Carlin et al. 2009), recent research shows far more diversity within this class of sugars (Kleikamp et al. 2020). Intriguingly, *C. thermarum* TA2.A1 also has ccmL and ccmM (Fig. 4), genes used for inorganic carbon concentration and capture (Ludwig et al. 2000). Capturing inorganic carbon should be wholly useless for a heterotrophic, aerobic organism such as *C. thermarum* TA2.A1, these systems are usually found in photosynthetic organisms. Considering this, its presence could originate from a more ancient environment, one in which the ability to scavenge any carbon, organic or inorganic, was a prime competitive advantage.

Iron sequestering is an extreme challenge in an alkaline environment with iron being almost totally insoluble. *C. thermarum* TA2.A1 has been reported to produce a siderophore composed of catecholate and hydroxamate (McMillan et al. 2010) chemical groups, but unfortunately this molecule could not be isolated. Here, we find the presence of a bacillibactin-like exporter, which is a catacholate siderophore, giving insight into new approaches for siderophore purification. Unsurprisingly, *C. thermarum* TA2.A1 has multiple siderophore import mechanisms to gather siderophores produced by other microbes (see Fig. 4). Lastly, *C. thermarum* TA2.A1 also has FAD/FMN export machinery (yeeO), the purpose of this is totally unknown.

### Central carbon metabolism

**Fig. 5:**
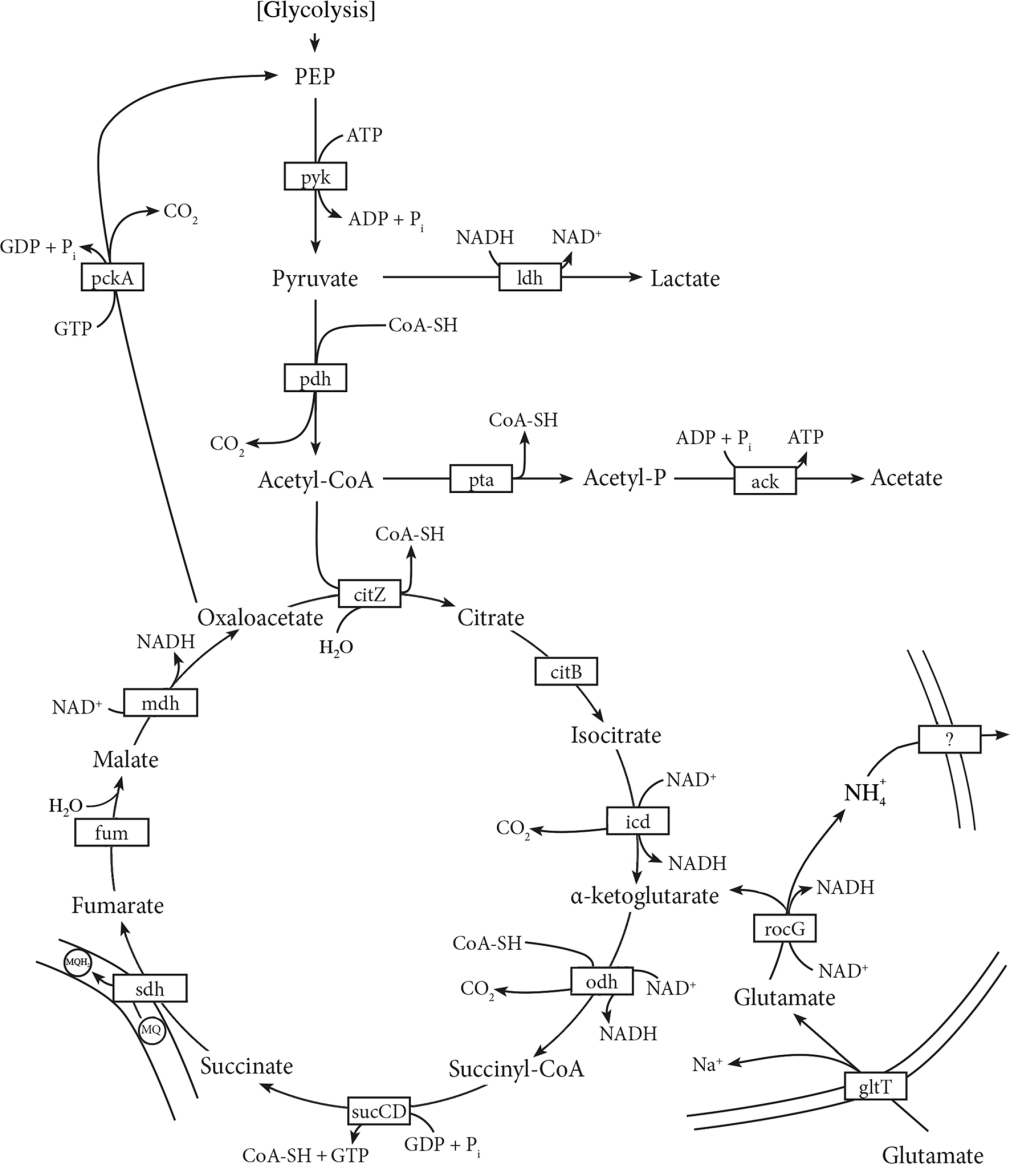
Proposed catabolic model for *C. thermarum* TA2.A1. The model consists of an Embden–Meyenhoff–Parnas type glycolysis (see Figure S1 for details), and a tricarboxylic acid (TCA) cycle, including a one-step shunt to connect glutamate to the TCA cycle. It also shows fermentative routes towards acetate and lactate. Furthermore, the glutamate uptake system is shown, and a pathway to alkalize local environment using NH_4_^+^. The parallel lines indicate membrane-bound proteins; MQ/MQH_2_ is the membrane-bound electron carrier menaquinone used in the electron transfer chain (see Fig. 4)

*Caldalkalibacillus thermarum* TA2.A1 is an aerobic, chemoheteroorganotrophic organism, preferably growing on glutamate or sucrose, which are mainly converted to CO_2_ and acetate (McMillan et al. 2009). After substrate import, *C. thermarum* TA2.A1 has a relatively straightforward catabolism consisting of the glycolysis and the tricarboxylic acid (TCA) cycle (see Figs. S3 and 5). For classical glycolytic sucrose consumption, the compound is imported via a phosphotransferase system, but in *C. thermarum* TA2.A1 symport is the dominant mechanism (Peddie et al. 2000) (see Fig. 4), and performs the split into fructose and glucose thereafter (Fig. S3). Glutamate catabolism in *C thermarum* TA2.A1 is intriguing, as it seems to differ from known pathways (Buckel and Barker 1974; Buckel 2001). These pathways include the strictly anaerobic mesaconate pathway first described in *Clostridium tetanomorphum* (Barker 1961), a pathway via 2-hydroxyglutarate discovered in *Peptostreptococcus asaccharolyticus* (Whiteley 1957) and a variation on the pathway via 2-hydroxyglutarate found in *Fusobacterium nucleatum* (Gharbia and Shah 1991). *C. thermarum* TA2.A1 lacks the genes for those pathways and instead we hypothesize it feeds glutamate directly into the TCA cycle via a one-step catalyzed by glutamate dehydrogenase (Fig. 5), an enzyme that confusingly enough has glutamate deaminase activity (Crozier et al. 1987).

**Fig. 6:**
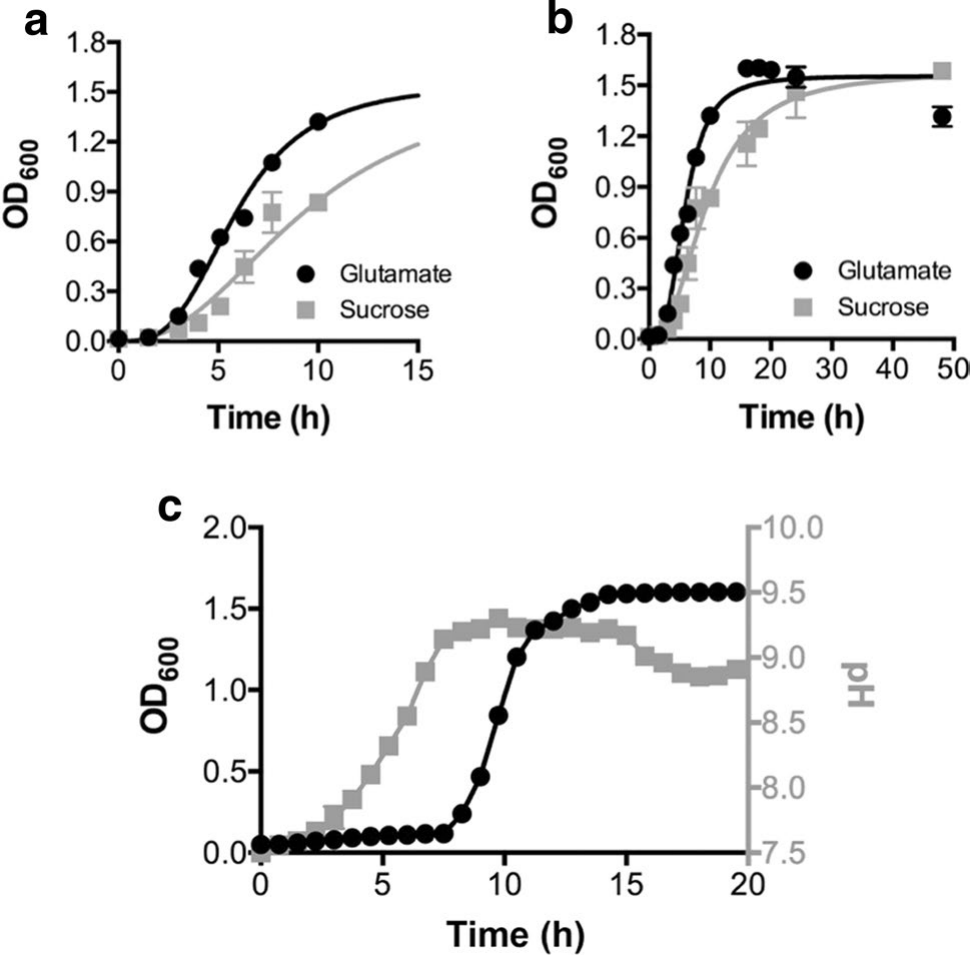
*C. thermarum* TA2.A1 growth on glutamate vs. sucrose and influence of starting pH. Effect of carbon source on the growth of *C. thermarum* TA2.A1 in flask batch-culture using alkaline basal medium supplemented with either 10 g L^−1^ l-glutamate or sucrose. In A and B the initial starting pH was 9.5 where as in C it was 7.5. **a** Growth over the first 10 h of culturing; **b** Full growth curves; **c** Growth on glutamate with initial pH at 7.5 showing a pH shift before onset of growth. The values reported are the means of four replicate experiments with the standard error of the means shown

Entering the TCA cycle via α-ketoglutarate will require some production of acetyl-CoA via gluconeogenesis to circumvent the problem of the unconventional TCA cycle access point. Being an aerobic organism, excess reducing equivalents are produced during dissimilation and these are respired in the ETC (see Fig. 4). However, nothing in the central metabolism suggests why *C. thermarum* TA2.A1 growth using glutamate as a carbon source has a consistently shorter lag phase and grows ~ 30% faster than when using sucrose (Fig. 6a-b), especially since both carbon sources are imported using ΔNa^+^-driven symport (Fig. 4). In addition, sucrose should theoretically yield more energy/mol since 2 rounds of glycolysis and 4 rounds of the TCA is possible (Fig. 5 and Fig. S3). It is noted that this is not due to a change in cell size, since the dry weight also has a ~ 30% higher mass when *C. thermarum* TA2.A1 is grown on glutamate after 16 h (Fig. S4).

We propose that the answer does not lie in the substrate that yields the most energy, but the substrate that allows another physiologically beneficial function. Therein we suggest two phenomena are likely at work. Firstly, the discrepancy between the onsets of growth could be due to the bacterial variety of the ‘resource allocation theory (Erickson et al. 2017)—fewer enzymes have to be expressed for glutamate consumption (Fig. 5), compared to sucrose (Fig. S3 and Fig. 5), and thus growth with this substrate may be kinetically advantageous. Secondly, the glutamate deamination into α-ketoglutarate by glutamate dehydrogenase releases ammonium, which has a p*K*_*a*_ of 9.25. This tends not to matter under alkaline conditions in a hot-pool, but when grown in a sealed volume, this does matter, as metabolic by-products are not washed away. Interestingly, we observe a longer lag-phase when grown on glutamate at pH 7.5 (Fig. 6c) that does not occur when grown on sucrose (McMillan et al. 2009). Upon onset of eventual growth, retesting pH shows a significant increase in extracellular pH. In short, deamination of glutamate is an excellent example of an extremophile modulating environmental conditions to suit its own requirements and to outcompete possible competitors. This allows alkaliphiles to be found in a number of non-alkaliphilic environments (Horikoshi 2004).

### Oxidative phosphorylation

In the electron transport chain an unexpectedly extensive variation in available respiratory enzymes is found with seeming redundancy (see Fig. 4). Since only menaquinone biosynthetic machinery could be identified (see Fig. S5), it is highly likely that all the enzymes involved in membrane-bound electron transport are reliant on menaquinone for physiological function (Fig. 4). *C. thermarum* TA2.A1 has both putative type I (ndh1) and characterized type II (ndh2) NADH dehydrogenases (Heikal et al. 2014; Godoy-Hernandez et al. 2019) (Fig. 4). The ndh1 is likely capable of translocating ions over the membrane, presumably protons, which would be used by the proton-linked F_1_F_o_ ATP synthase (McMillan et al. 2007). Using a proton-translocating variant of NADH dehydrogenase yields more energy, begging the question why the microorganism has an ndh2 as well. Reports into the physiological function of the type II NADH dehydrogenase has yielded various explanations from simply balancing NADH/NAD^+^ concentrations (Rao et al. 2008), to responses to changing oxygen levels (Melo et al. 2004). In agreement with membrane measurements (McMillan et al. 2009), a putative sdh1 was identified (see Fig. 4) which is capable of the succinate to fumarate conversion, however any fumarate reductase activity of this enzyme is unknown. In the previous genome an operon annotated as a cytochrome *b*_6_*f* was annotated. However, while genes indeed include a cytochrome *b*_6_, there is no homology to a cytochrome *f*, but there is to a cytochrome *c*_1_ (Fig. S6). This leads us to believe that this organism has a cytochrome *b*_6_*c*_1_, which is a novel-hybrid type complex III also found in *G. stearothermophilus* (Sone et al. 1996). Curiously, the iron-sulfur cluster of *C. thermarum* TA2.A1′s cyt.*b*_6_*c*_1_ also shows similarities to the iron-sulfur cluster in cyt.*b*_6_*f* of the cyanobacterium *Synechocystis sp.* PCC 6803 (Fig. S7), and may be an evolutionary precursor. Supporting this proposition, while a *b*_6_*c*_1_ has not been examined, a synthetically constructed *b*_6_*c*_1_ was constructed and was able to functionally replace a cytochrome *b*_6_*f* in *Rhodobacter capsulatus* in the process of cellular photosynthesis (Saribas et al. 1999).

At the electron-accepting end of the respiratory chain, *C. thermarum* TA2.A1 has extreme plasticity. Four terminal oxidases were identified; cytochromes *aa*_3_, *ba*_3_, *bb*_3_, and *bd* (see Fig. 4). In cytochromes *aa*_3_, *ba*_3_ and *bb*_3_ *C. thermarum* TA2.A1 has three types of proton-translocating terminal oxidases. Previous reports have identified that these enzymes pump different amounts of protons per oxygen molecule reduced (Jones et al. 1975; Reynafarje et al. 1982). This, together with a non-proton pumping cytochrome *bd* leads to the notion that the variation is there for extreme optimization since *C. thermarum* TA2.A1 lives in an extremely proton-poor environment and that membrane potential is likely playing a greater role than pH for oxidative phosphorylation. Lastly, *C. thermarum* TA2.A1 has an extensively studied (Cook et al. 2003; Keis et al. 2004, 2006; Stocker et al. 2005; Meier et al. 2007) proton-coupled F_1_F_o_-type ATP synthase (see Fig. 4) which is only capable of ATP synthesis, but not hydrolysis (McMillan et al. 2007, 2016). The operon has previously been shown to have the typical canonical subunits and atpI, but here we also report the presence of atpZ. The functions of atpI and atpZ are unknown, and although both have been proposed to link to magnesium transport (Hicks et al. 2003), no concrete biochemical assays have been conducted to verify this proposal.

Although not membrane bound, it is indeed curious in the context of the link to plant evolution that we also found an electron bifurcating enzyme of the *fix* class (see Fig. 4). This enzyme transfers electrons from NADH to ferredoxin, and is generally found in plant-associated microbes (Ledbetter et al. 2017). In the context of *C. thermarum* TA2.A1 the use of ferredoxin may be involved in glutamate synthesis from glutamine and α-ketoglutarate (van den Heuvel et al. 2003), giving another possible reason why the microorganism grows so well on this substrate (i.e. it does not have to synthesize glutamate because it is bioavailable).

### Ion homeostasis

An important feature of *C. thermarum* TA2.A1 is that while it is indeed an obligate alkaliphile when grown on C4-dicarboxylates, it is a facultative alkaliphile when grown on fermentable substrates such as sucrose. This naturally means *C. thermarum* TA2.A1 has to be able to adapt to neutral pH conditions in which more protons are located outside of the cell than in (Krulwich and Guffanti 1989), and an inverted ΔpH at alkaline conditions (Yumoto 2002). Interestingly enough, *C. thermarum* TA2.A1 does this without altering its maximum specific growth rate, propping up the dearth of its proton motive force—a decline of − 164 mV at pH 7.5 to − 78 mV at pH 10—with a − 100 mV sodium motive force (Olsson et al. 2003). Since the F_1_F_O_-type ATP synthase is only capable of importing protons, and cytoplasmic pH is maintained between pH 7.8–8.5, we envision a substantial role for monovalent cation antiporters. This view is strengthened by an earlier hypothesis describing that antiport of proton vs. potassium/sodium contributes to energy generation via the ETC in alkaliphiles (Krulwich et al. 1994). Indeed, to maintain cytoplasmic pH at pH 9.5 we identified nhaC and mrp for Na^+^ extrusion and H^+^ uptake (see Fig. 4). However, to maintain cytoplasmic pH at pH 7.5 we identified a cation antiporter (nhaP) capable of importing K^+^, and possibly Na^+^, at the cost of exporting H^+^ (Fig. 4). If the balance of cations is wholly disrupted, H^+^ export is undesirable, or H^+^ import impossible, *C. thermarum* TA2.A1 has ‘emergency override systems’. ATP-dependent uniporters for Na^+^ (natCAB) and K^+^ (pstS) are present for such a situation (see Fig. 4).

### Regulatory systems

*Caldalkalibacillus thermarum* TA2.A1 has three annotated toxin-antitoxin systems. Toxin/antitoxin systems are generally regarded as ‘selfish genes’ deriving from plasmids. It has been proposed that if after replication the plasmid is absent in the daughter cell, the toxin-antitoxin system promotes its own survival over the survival of the cell as a whole (Guglielmini and van Melderen 2011). When integrated into a bacterial genome, they have also been shown to regulate translation as a response to environmental conditions, having some bearing on cell metabolism and cell death (Hayes and Van Melderen 2011). Taking this into account, the *loci* of the annotated toxin-antitoxin systems are intriguing. The toxin-antitoxin mazEF system was found just downstream of the operons for ndh1 and the F_1_F_O_-ATP synthase. A second toxin-antitoxin ndoAI/ndoA system (Wu et al. 2011) was revealed by this new genome and found upstream of an operon encoding for tRNA’s for asparagine, serine, glutamate, aspartate, glutamine and leucine. The third system, a doc toxin/antitoxin system (death on curing) is located just upstream of a few transporters, including that of the C4-dicarboxylates. We hypothesize that these systems might regulate expression, of for instance oxidative phosphorylation (mazEF), when environmental conditions become adverse, or even program cell-death of a subpopulation (Engelberg-Kulka et al. 2006).

## Conclusion

This article outlines the first report of a complete circular chromosome of the thermoalkaliphile *Caldalkalibacillus thermarum* TA2.A1. Considering the increase in genetic data of 15.5% over the previously available data, we decided to perform an in-depth analysis of its features and placement within a novel order. Consequently, we give a hypothesis for a putative origin region and we outline similarities to plant genomes. These similarities include a cytochrome *b*_6_*c*_1_ complex that is a possible homolog of the plant cytochrome *b*_6_*f*, the similarity in F_1_F_O_-ATP synthase *c*-subunit rotor ring size, and the means to capture inorganic carbon. Furthermore, we outline a catabolic pathway via the oxidative TCA cycle, something that has not been reported yet, to the best of our knowledge. We also find interesting regulatory systems, such as CRISPRs and toxin/antitoxin systems, the latter of which could have a bearing on regulation of cellular processes, like oxidative phosphorylation. Finally, we describe how its many monovalent cation antiporters are capable of enabling the facultative alkaliphilic lifestyle of *C. thermarum* TA2.A1. These features are just a tip of the iceberg of new data made available by this updated genome, indicating the value of continuously re-sequencing genomes present in the NCBI database, as our sequencing methods are ever-improving.

## Electronic supplementary material

Below is the link to the electronic supplementary material.

Supplementary file 1 (PDF 7928 kb)
