# Supplementary material for: Genomic analysis of Caldalkalibacillus thermarum TA2.A1 reveals aerobic alkaliphilic metabolism and evolutionary hallmarks linking alkaliphilic bacteria and plant life
Source: Extremophiles. 2020 Oct 8;24(6):923–35. doi: 10.1007/s00792-020-01205-w (PMC7561548; doi:10.1007/s00792-020-01205-w)
Supplement: Supplementary file 1 — (PDF 7928 kb) [file 792_2020_1205_MOESM1_ESM.pdf]

Supplementary Information for: Genomic analysis of *Caldalkalibacillus thermarum* TA2.A1 reveals aerobic alkaliphilic metabolism and evolutionary hallmarks linking alkaliphilic bacteria and plant life

Samuel I. de Jong[1], Marcel A. van den Broek[1], Alexander Y. Merkel[2], Pilar de la Torre Cortes[1], Falk Kalamorz[3], Gregory M. Cook[4], Mark C.M van Loosdrecht[1], Duncan G.G. McMillan[1*]

[1]Department of Biotechnology, Delft University of Technology, Delft, The Netherlands

[2]Winogradsky Institute of Microbiology, Research Centre of Biotechnology, Russian Academy of Sciences, Moscow, Russia

[3]The New Zealand Institute for Plant and Food Research, Lincoln, New Zealand

[4]Department of Microbiology and Immunology, The University of Otago, Dunedin, New Zealand

Running title: The complete *Caldalkalibacillus thermarum* TA2.A1 genome

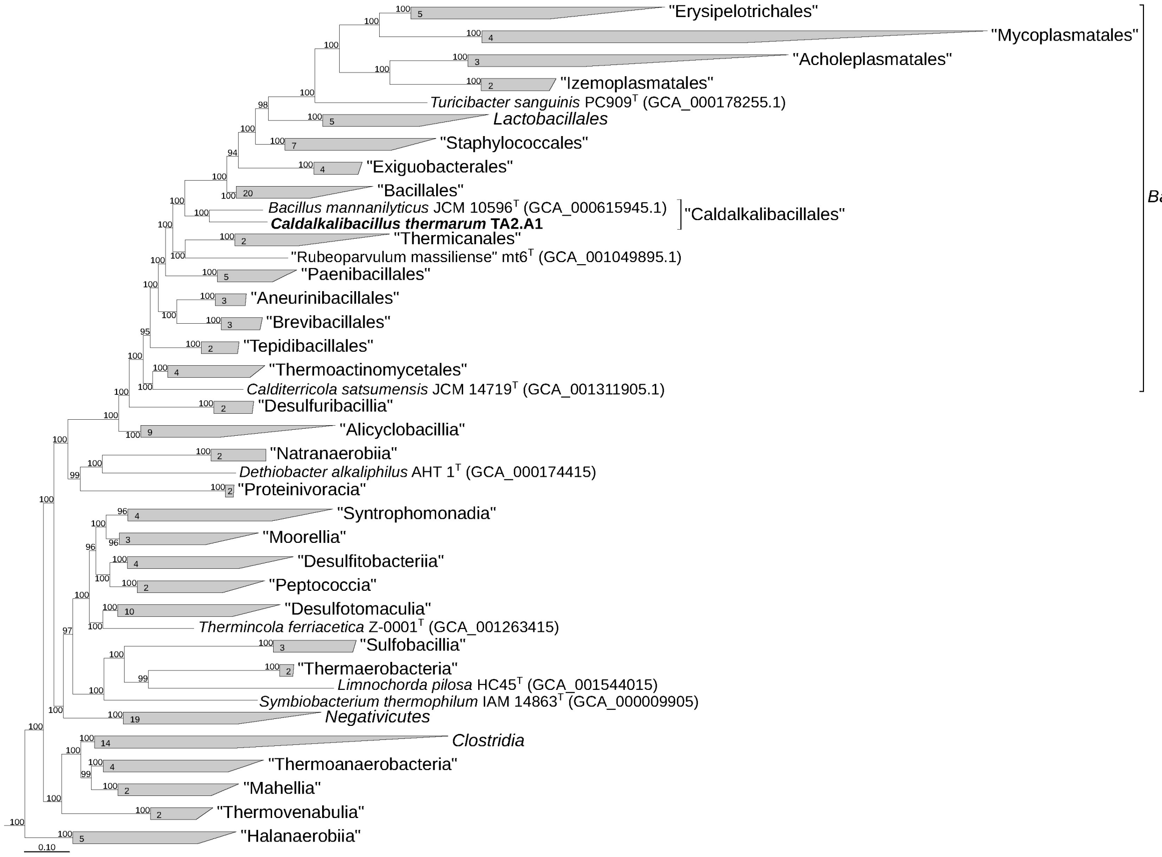

Figure S1. Full Phylogenetic tree describing the placement of *C. thermarum* TA2.A1 in a new order

Placement of *C. thermarum* TA2.A1 within the "Firmicutes" phylum based on phylogenetic analysis of concatenated partial amino acid sequences of 120 bacterial conservative proteins[40] by maximum likelihood inference; taxonomic designations correspond with Genome Taxonomy DataBase[40]. Bootstrap values are shown at the nodes. Bar, 0.10 changes per position.

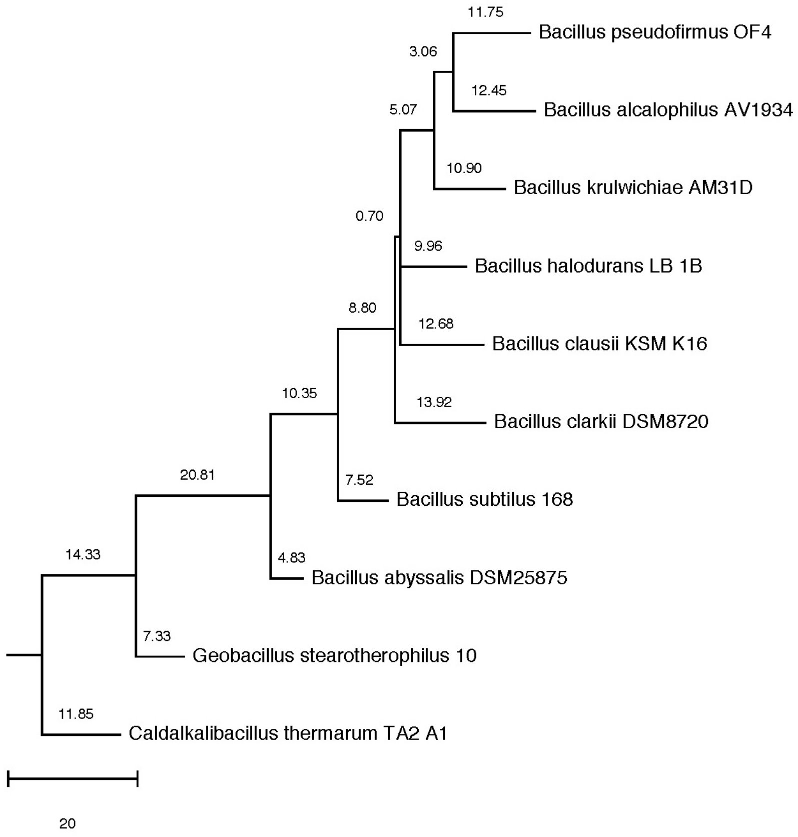

Figure S2. Whole genome SNP based phylogeny of Bacillus species
Reference free whole genome SNP based phylogeny of Bacillus species (with *E. coli* K-12 as outgroup; not shown) show that the strain *C. thermarum* TA2.A1 is a deeply rooted member of the Bacilli class.

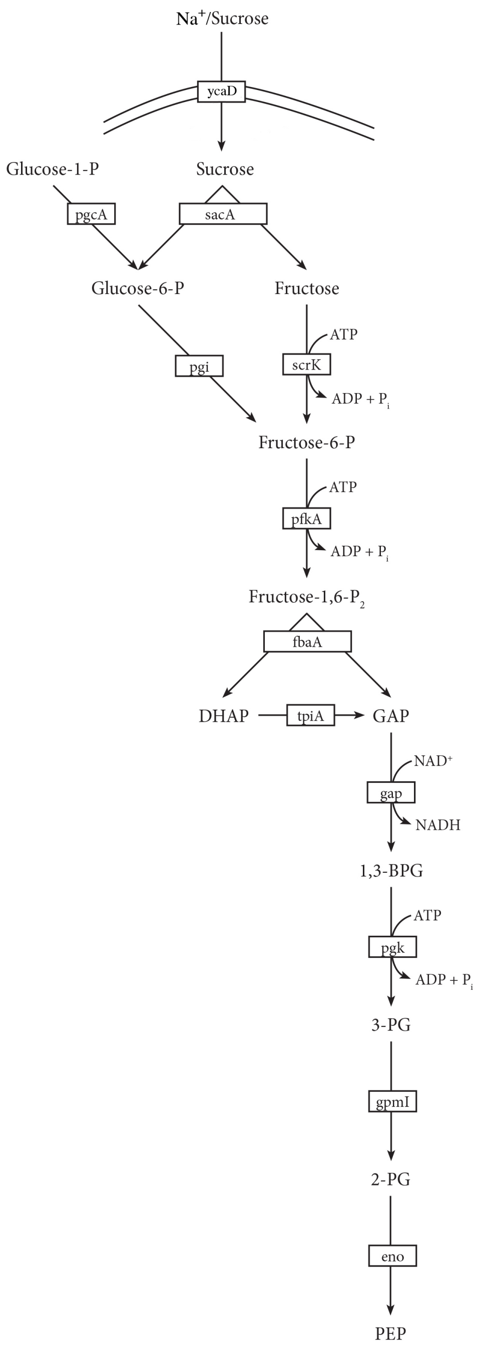

Figure S3. Glycolysis and sucrose import by symport in *Caldalkalibacillus thermarum* TA2.A1

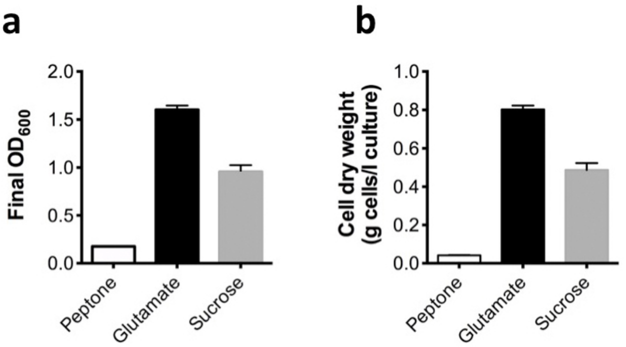

Figure S4. ***Caldalkalibacillus thermarum*** **TA2.A1 growth on glutamate vs sucrose**
Effect of carbon source on the growth of *C. thermarum* TA2.A1 in flask batch-culture using either alkaline basal medium containing trypticase peptone (no additional carbon source) or basal medium supplemented with either 50 mM Na-glutamate or sucrose. A, Final optical density (at 600 nm) of a culture grown for 16 hours to stationary phase. B, Dry weight of cells from the cultures in A. The values reported are the means of four replicate experiments with the standard error of the means shown.

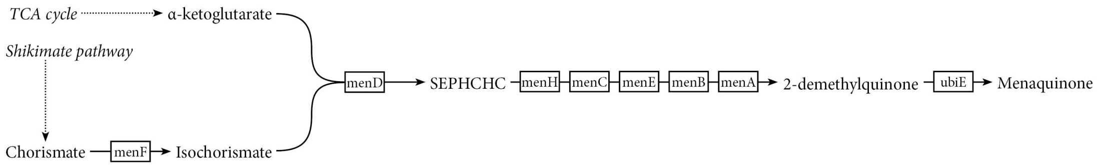

Figure S5. Menaquinone synthesis pathway in *Caldalkalibacillus thermarum* TA2.A1.
All genes are present in the genome, as well as the machinery for the TCA cycle and the Shikimate pathway.

```
Identities: 151/263 (57%)       Positives: 193/263 (73%)        Gaps 16/263 (6%)

Caldalkalibacillus thermarum TA2.A1  (2)     NHHKDVKYVEDSRILARRVPNIPKDYSEYPGKTEPFWPNFLLKEWMVGAVVLIGFLVLTV
                                             +  K +K+V DSR+ A R PNIPKDYSEYPGKTE FWPNFLLKEW+VG+V L+GFL LTV
Geobacillus stearothermophilus 10    (7)     HRGKGMKFVGDSRVPAVRKPNIPKDYSEYPGKTEVFWPNFLLKEWLVGSVFLVGFLCLTV

Caldalkalibacillus thermarum TA2.A1  (67)    AHEPPLERLADPTDTGYAPVPDWYFLFLYELLKYTYASGPYTVIGTVAIPGLAFAALLLA
                                             AH  PLER+ADPTDT Y P+PDWYFLFLY+LLKY+YASGPYTV+G + IPGLAF ALLLA
Geobacillus stearothermophilus 10    (62)    AHPSPLERIADPTDTTYVPLPDWYFLFLYQLLKYSYASGPYTVVGAIIIPGLAFGALLLA

Caldalkalibacillus thermarum TA2.A1  (127)   PWLDRSKERRPVKRPIATSIMLLVIASIFVLTYDAYKAHDWS----QNEVYAWDSPYFDV
                                             P+LDR  ERRP KRP+AT +MLL +A++  LT++A   HDW     Q ++ A      +V
Geobacillus stearothermophilus 10    (122)   PFLDRGPERRPWKRPVATGMMLLALAAMIYLTWEAVVTHDWKKAAEQGKIRA------EV

Caldalkalibacillus thermarum TA2.A1  (183)   EIDTSHPAYELYVQYTCINCHGENLEGGAAGVPLNLIGSELNKDEIKEVIINGQGSMPGG
                                             +IDT+   Y++    TC +CHG+NL GGA     +LIG+ L  +EI ++   G+G+MP G
Geobacillus stearothermophilus 10    (176)   KIDTNAEGYKIAQANTCTSCHGQNLSGGAGP---SLIGTGLKPEEIAKIAREGKGNMPPG

Caldalkalibacillus thermarum TA2.A1  (243)   LV--TDEEDLEALAEWLASLDGE  (263)
                                             +   TDEE L+ L+E++A L  E
Geobacillus stearothermophilus 10    (233)   VFKGTDEE-LKKLSEFIAGLKAE  (254)
```

Figure S6. Alignment of *Caldalkalibacillus thermarum* TA2.A1 and *Geobacillus stearothermophilus* cytochrome c_1 subunits.
Alignment was generated with the BLAST tool publicly available at https://blast.ncbi.nlm.nih.gov/.

```
Identities: 45/170 (26%)        Positives: 66/170 (68%)        Gaps: 35/170 (20%)

Caldalkalibacillus thermarum TA2.A1  (9)     VSRRQFLNYTLMGVGGFLVSATITPMIRFAIDPVLKVGEDREMVPVGDISEFGKEYKRVD
                                             + RRQF+N    G    + +  + P +++ I P            V      G + K
Synechocystis sp. PCC 6803          (13)     LGRRQFMNLLTFGTITGVAAGALYPAVKYLIPPSSG----GSGGGVTAKDALGNDVKV--

Caldalkalibacillus thermarum TA2.A1  (69)    FKLLIKDGWTEYERLYSAWVRVMDNG--------EVQ--------ALSPVCTHLGCTVQW
                                                      TE+   ++A  RV+  G         VQ         ++ VCTHLGC V W
Synechocystis sp. PCC 6803           (67)    ---------TEFLASHNAGDRVLAQGLKGDPTYIVVQGDDTIANYGINAVCTHLGCVVPW

Caldalkalibacillus thermarum TA2.A1  (113)   DTHDDYPNHFFCPCHDGLYDENGINIPGTPPTRPLDVYEYEVGEDGKLYL  (162)
                                             +  +   N F CPCH   Y+  G  + G  P   L +    V +D KL L
Synechocystis sp. PCC 6803           (118)   NASE---NKFMCPCHGSQYNAEGKVVRGPAPLS-LALAHATVTDDDKLVL  (163)
```

Figure S7. Alignment of *Caldalkalibacillus thermarum* TA2.A1 and *Synechocystis sp.* PCC 6803 iron-sulfur cluster subunits incorporated in cyt.b_6c_1 and cyt.b_6f respectively.
Alignment was generated with the BLAST tool publicly available at https://blast.ncbi.nlm.nih.gov/.
